# A new predictive parameter for dose‐volume metrics in intensity‐modulated radiation therapy planning for prostate cancer: Initial phantom study

**DOI:** 10.1002/acm2.14250

**Published:** 2023-12-25

**Authors:** Yuki Saito, Ryusuke Suzuki, Naoki Miyamoto, Kenneth Lee Sutherland, Takahiro Kanehira, Masaya Tamura, Takashi Mori, Kentaro Nishioka, Takayuki Hashimoto, Hidefumi Aoyama

**Affiliations:** ^1^ Graduate School of Biomedical Science and Engineering Hokkaido University Sapporo Japan; ^2^ Department of Medical Physics Hokkaido University Hospital Sapporo Japan; ^3^ Faculty of Engineering Hokkaido University Sapporo Japan; ^4^ Global Center for Biomedical Science and Engineering Faculty for Medicine Hokkaido University Sapporo Japan; ^5^ Department of Radiation Oncology Hokkaido University Hospital Sapporo Japan; ^6^ Department of Radiation Oncology Faculty of Medicine Hokkaido University Sapporo Japan

**Keywords:** intensity‐modulated radiation therapy, overlap volume, pinnacle^3^ Auto‐Planning, plan quality, prostate cancer, step and shoot, volumetric modulated arc therapy

## Abstract

**Background:**

Organ‐at‐risk (OAR) sparing is often assessed using an overlap volume‐based parameter, defined as the ratio of the volume of OAR that overlaps the planning target volume (PTV) to the whole OAR volume. However, this conventional overlap‐based predictive parameter (COPP) does not consider the volume relationship between the PTV and OAR.

**Purpose:**

We propose a new overlap‐based predictive parameter that consider the PTV volume. The effectiveness of proposed overlap‐based predictive parameter (POPP) is evaluated compared with COPP.

**Methods:**

We defined as POPP = (overlap volume between OAR and PTV/OAR volume) × (PTV volume/OAR volume). We generated intensity modulated radiation therapy (IMRT) based on step and shoot technique, and volumetric modulated arc therapy (VMAT) plans with the Auto‐Planning module of Pinnacle^3^ treatment planning system (v14.0, Philips Medical Systems, Fitchburg, WI) using the American Association of Physicists in Medicine Task Group (TG119) prostate phantom. The relationship between the position and size of the prostate phantom was systematically modified to simulate various geometric arrangements. The correlation between overlap‐based predictive parameters (COPP and POPP) and dose‐volume metrics (mean dose, V_70Gy_, V_60Gy_, and V_37.5 Gy_ for rectum and bladder) was investigated using linear regression analysis.

**Results:**

Our results indicated POPP was better than COPP in predicting intermediate‐dose metrics. The bladder results showed a trend similar to that of the rectum. The correlation coefficient of POPP was significantly greater than that of COPP in < 62 Gy (82% of the prescribed dose) region for IMRT and in < 55 Gy (73% of the prescribed dose) region for VMAT regarding the rectum (*p* < 0.05).

**Conclusions:**

POPP is superior to COPP for creating predictive models at an intermediate‐dose level. Because rectal bleeding and bladder toxicity can be associated with intermediate‐doses as well as high‐doses, it is important to predict dose‐volume metrics for various dose levels. POPP is a useful parameter for predicting dose‐volume metrics and assisting the generation of treatment plans.

## 1 INTRODUCTION

Recently, intensity‐modulated radiation therapy (IMRT) and volumetric modulated arc therapy (VMAT) have become increasingly prevalent in radiation therapy for prostate cancer. These techniques deliver improved dose conformity to the target while minimizing the dose to surrounding organs at risk (OARs). For radiation‐induced toxicities in prostate cancer, it is well‐known that rectal bleeding is associated with high dose (70−80 Gy).^1^, ^2^, ^3^ On the other hand, studies have reported that rectal bleeding is related to the volume receiving an intermediate dose (V_30Gy_–V_60 Gy_).^4^, ^5^, ^6^, ^7^, ^8^ In addition, researchers have reported that bladder toxicity is associated with high dose (70−78 Gy)^9^, ^10^, ^11^, ^12^ and intermediate dose (V_14Gy_–V_40 Gy_).^13^, ^14^ To reduce the risk of developing radiation‐induced toxicities in prostate cancer, the dose to the rectum and bladder should be lowered as much as possible.

In contrast to conventional radiotherapy, the planner's skill greatly influences the treatment plan quality of an IMRT plan. Inverse optimization approaches used in IMRT planning require a large amount of manual effort.^15^, ^16^ To lower the OAR dose as much as possible, recent studies have discussed the effectiveness of the overlap ratio, which is the ratio of overlap between an OAR and the planning target volume (PTV).^17^, ^18^, ^19^ Moore et al. reported that the achievable OAR sparing is related to the overlap ratio.^20^ They proposed a predictive model for the OAR mean dose using the overlap ratio. Their results indicated that by using the predictive model, the OAR mean dose was lowered, and the interplanner treatment plan variability was reduced by incorporating the predictive model into the planning workflow. Chao et al. investigated the correlation between the overlap ratio and dose‐volume metrics such as V_75Gy_, V_70Gy_, V_60Gy_, and V_40Gy_ for the rectum.^21^ Their results also indicated that the overlap ratio was beneficial for predicting OAR sparing. Nevertheless, the weakness of the overlap ratio (which we call the conventional overlap‐based predictive parameter [COPP] in this paper) is that the PTV volume is not considered. Thus, the COPP cannot distinguish different PTV sizes. Two cases with the same overlap ratio and different PTV volumes have the same COPP values, despite the different OAR doses. This means that the COPP cannot predict the OAR dose with distinction of PTV volume. In practice, OAR doses are dependent on the PTV size and intuitively increase with the PTV size.

In this study, we hypothesize that the prediction of dose‐volume metrics using the COPP will be improved by considering the relationship between the OAR volume and the PTV volume. Based on this hypothesis, we proposed overlap‐based predicted parameter in this study. The effectiveness of proposed overlap‐based predictive parameter (POPP) is demonstrated with simulations for IMRT and VMAT in several geometry cases, in which the PTV and/or OAR positions and sizes were systematically varied using the American Association of Physicists in Medicine Task Group‐119 (TG119) prostate phantom.

## 2 MATERIALS AND METHODS

### 2.1 Definition of the predictive parameters

First, we define the COPP, which has been used for predicting OAR dose in recent studies^17^, ^18^, ^19^:

(1) $$COPP\;=\;\frac{V_{OV}}{V_{OAR}}$$

where *V_OV_* is the overlap volume of OAR and PTV and *V_OAR_* is the volume of OAR.

To consider the volume relationship between PTV and OAR, we propose the following predicted parameter, POPP:

(2) $$POPP\;=\;\frac{V_{OV}}{V_{OAR}}\times \frac{V_{PTV}}{V_{OAR}}$$

where *V_PTV_* is the volume of the PTV. The POPP is defined as the product of the COPP and the ratio of PTV volume to OAR volume. We propose this parameter based on the assumption that the OAR dose should increase as the PTV volume becomes larger and the OAR volume becomes smaller.

### 2.2 Dosimetric simulations

We investigated the effectiveness of the POPP in two simulations. We used the TG119 prostate phantom in these simulations. The structure sets were expanded, referring to clinical studies,^22^, ^23^ since the structures were smaller than in the actual patients.

#### 2.2.1 Simulation 1: Cases with two PTV sizes with the same overlap ratio

**FIGURE 1:**
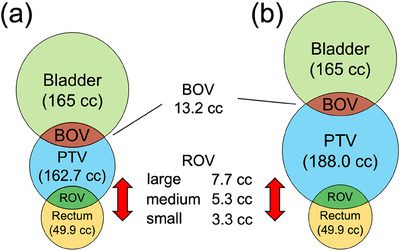
Schematic diagram of phantom geometries for (a) small PTV volume and (b) large PTV volume. The three types of ROV are small, medium, and large. RV and BV were kept constant in all cases. BOVs were kept constant in all cases by shifting the bladder structure. BOV, bladder overlap volume; BV, bladder volume; PTV, planning target volume; ROV, rectum overlap volume; RV, rectum volume.

In the first simulation, we studied two PTV cases that had the same overlap ratio. This was done in order to evaluate the effectiveness of the POPP in a situation in which the COPP cannot distinguish PTV size. Figure 1 show a schematic diagram of the phantom setup in this simulation for a small PTV case and a large PTV case, respectively. PTV volumes were set at 162.7 and 188.0 cc, respectively. We set the rectum volume (RV) and bladder volume (BV) in this simulation at 49.9 and 165.0 cc, respectively. In this simulation, we studied three values of the rectum overlap volumes (ROV), which is the overlap volume of the rectum and PTV. To set the same COPP value with different PTV sizes, we set the ROV at 3.3, 5.3, and 7.7 cc for both PTV cases. We set the bladder overlap volume (BOV), which is the overlap volume of the bladder and PTV, at 13.2 cc in this simulation. Dosimetric simulation was conducted in the six cases that consisted of the combinations of two PTV sizes and three ROV values.

#### 2.2.2 Simulation 2: Various rectum and bladder geometries

**FIGURE 2:**
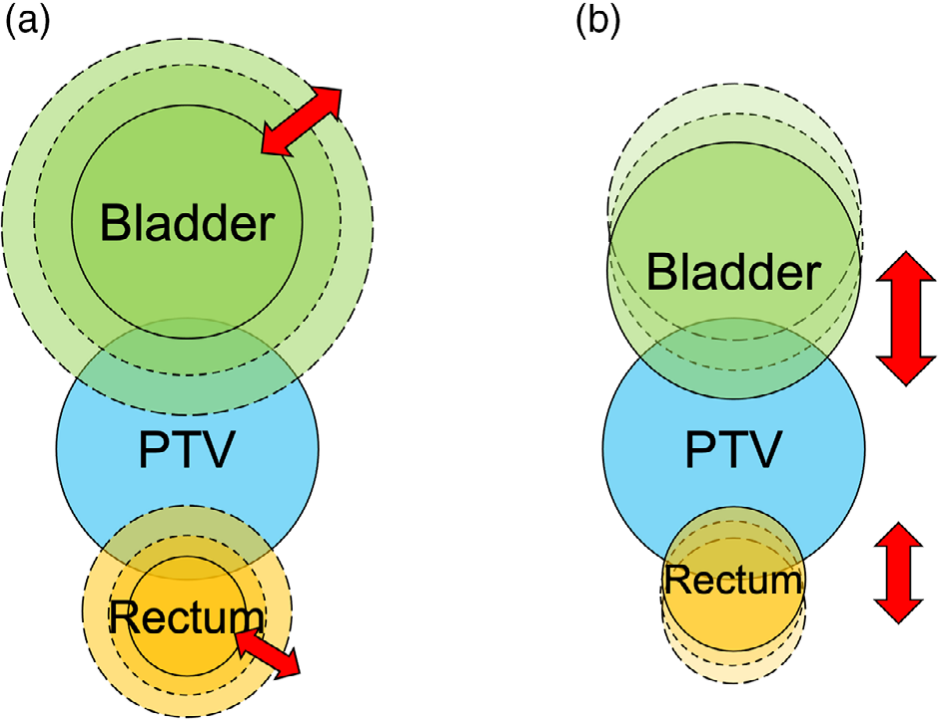
Schematic diagram of phantom geometries in simulation 2. (a) Volume change in the rectum and bladder. (b) The shift of the rectum and bladder in the AP direction. The PTV volume and position were fixed. AP, anterior–posterior; PTV, planning target volume.

For this simulation, we investigated the effectiveness of POPP compared with COPP in several phantom setups by systematically varying the volumes and positions of the rectum and bladder while considering various patient anatomies. Figure 2 shows a schematic diagram of the phantom setup in simulation 2. Figure 2a shows the volume change in rectum and bladder. The rectum was expanded from the original phantom size by 4, 5, and 6 mm in the AP and LR directions. The corresponding RV values were 42.5, 50.0, and 58.2 cc. The bladder was expanded from the original phantom size by 7, 10, and 12 mm in all directions. The corresponding BV values were 124.2, 165.0, and 197.0 cc. The PTV was expanded from the original phantom size by 16 mm in the AP and LR directions, resulting in 162.7 cc. The PTV volume and PTV position were fixed in this simulation because we were interested in the relative geometrical relationship between the PTV and OARs. Figure 2b shows the position shift of the rectum and bladder in the AP direction. The rectum position was shifted from the phantom original position by 10, 12, and 14 mm in the posterior direction. The bladder position was shifted from the original phantom position by 5, 8, and 11 mm in the anterior direction. We studied a total of 81 cases with combinations of nine rectum configurations (three volumes and three positions cases) and nine bladder configurations (three volumes and three positions cases).

### 2.3 Treatment planning

IMRT plans with seven fields (gantry angles: 0°, 51°, 102°, 153°, 207°, 258°, 309°) and VMAT plans with two coplanar full arcs were generated for each case described above using the Pinnacle^3^ treatment‐planning system (v14.0, Philips Medical Systems, Fitchburg, WI). We used the Auto‐Planning module to exclude the dependence on the planner's skill. Auto‐Planning is the function implemented in the Pinnacle^3^, which automatically optimize the treatment plan according to the user‐specified dose goals. In the Auto‐Planning optimization process, the following consecutive loop is repeated; (1) the ROIs to improve the target coverage and OARs sparing are automatically created,^24^ (2) the objectives for these ROIs are automatically established based on the specified dose goals, (3) the weights for their objectives are automatically determined, and (4) optimization is performed. Nawa et al. reported that the treatment plans generated by Auto‐Planning could be comparable or better with less interplanner variation as compared with plans created by human planners.^24^ All plans were created for Varian Clinac iX equipped with Millennium 120‐leaf multileaf collimators using 6‐MV photons. The prescription dose for the PTV was set to 75.6 Gy, and all plans required at least 95% of the volume of the PTV receiving the prescription dose. All plans followed the TG119 dose goals: V_75Gy_ < 10% and V_70Gy_ < 30% for the rectum and bladder. Table 1 lists the optimization goals for the Auto‐Planning used for all simulations.

**TABLE 1:** Dose optimization goals.

Target optimization goals		
ROI	Dose	
PTV	7800 cGy	

Optimization goals for target and organs at risk		
ROI	Goal	Priority/Compromise
PTV	D_max_ < 7636 cGy	High/yes
Rectum	V_75Gy_ < 10%	High/no
Rectum	V_70Gy_ < 30%	High/no
Bladder	V_75Gy_ < 10%	Medium/yes
Bladder	V_70Gy_ < 30%	Medium/yes
ring	D_max_ < 4536 cGy	Medium/yes

### 2.4 Plan evaluation and regression analysis

We evaluated the generated plans in terms of conformity number (CN)^25^ and homogeneity index (HI).^26^ CN is defined as CN = (*TV_PIV_*/*TV*)×(*TV_PIV_*/*PIV*), where $TV_{PIV}$ represents the volume of PTV receiving more than the prescription dose, TV represents the target volume, and PIV represents the volume receiving more than the prescription dose. HI is defined as HI = (D_2_ − D_98_)/D_50_, where D_2_, D_50_, and D_98_ are the minimum dose covering 2%, 50%, and 98% of the target volume, respectively.

We investigated the correlations between the predictive parameters (COPP or POPP) and the dose‐volume metrics using linear regression analysis. In this study, the dose‐volume metrics include mean dose (D_mean_), V_70Gy_, V_60Gy_, and V_37.5 Gy_ for the rectum and bladder.

Tests of statistical significance between the correlation coefficient of COPP and that of POPP were performed by paired‐samples *t*‐test at a 5% significance level.

## 3 RESULTS

### 3.1 Linear regression analysis in simulation 1

**FIGURE 3:**
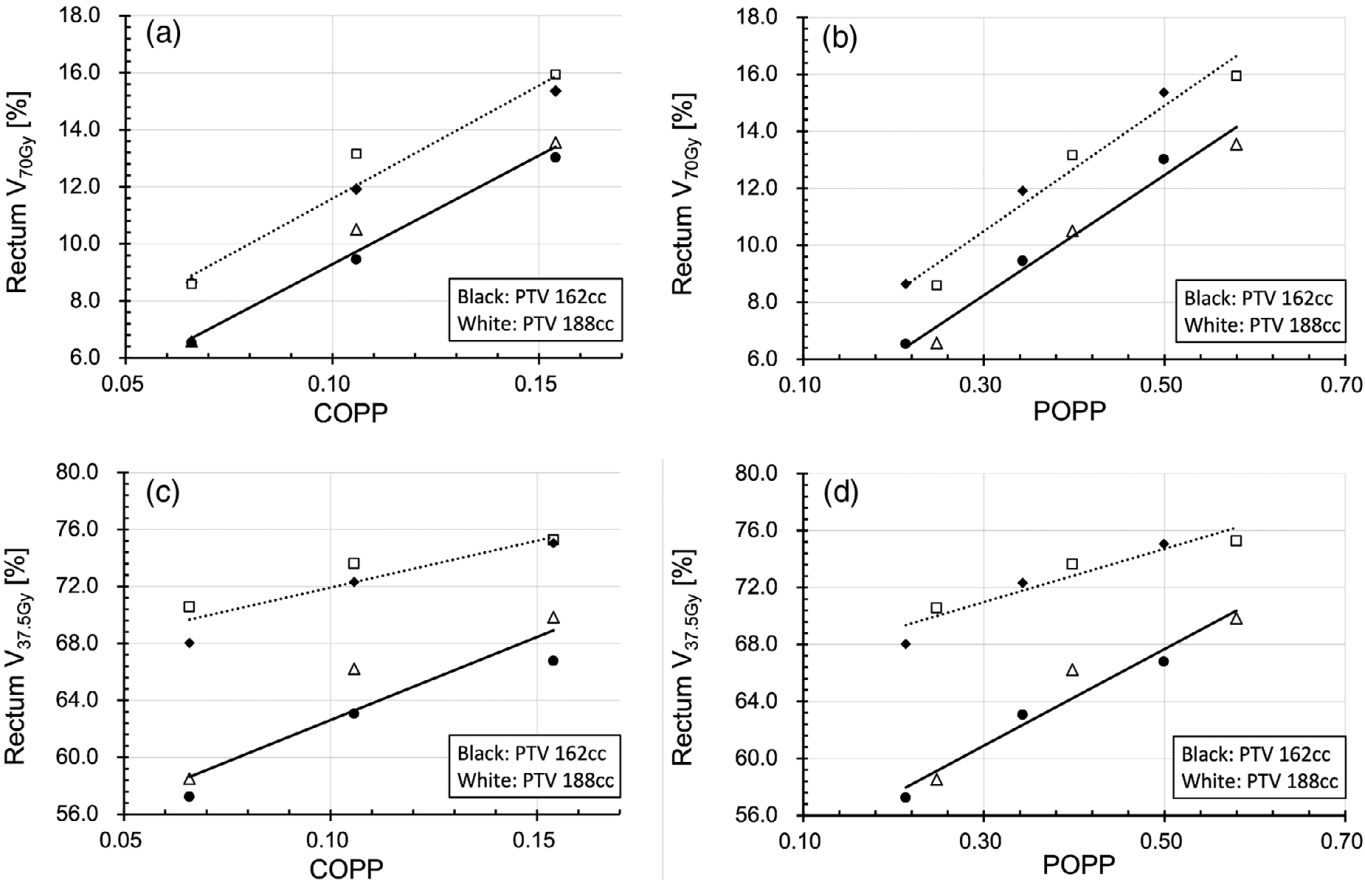
Linear regression analysis of the overlap‐based predictive parameter for rectum V_70Gy_ and rectum V_37.5 Gy_ with different PTV volumes for IMRT (dashed) and VMAT (solid): (a) rectum V_70Gy_ against COPP, (b) rectum V_70Gy_ against POPP, (c) rectum V_37.5 Gy_ against COPP, and (d) rectum V_37.5 Gy_ against POPP. Three different ROV patterns were used for two types of PTV volume, whereas the RV, BV, and BOV were kept constant. Data are plotted for two types of PTV volume: small for IMRT (black diamond), large for IMRT (white square), small for VMAT (black circle), and large for VMAT (white triangle). BOV, bladder overlap volume; BV, bladder volume; COPP, conventional overlap‐based predictive parameter; IMRT, intensity modulated radiation therapy; POPP, proposed overlap‐based predictive parameter; PTV, planning target volume; ROV, rectum overlap volume; RV, rectum volume; VMAT, volumetric modulated arc therapy.

Figure 3 shows linear regression analysis of the overlap‐based predictive parameter for rectum V_70Gy_ and rectum V_37.5 Gy_ with different PTV volumes for IMRT and VMAT in simulation 1. Figure 3a,c show the dose‐volume metrics (V_70Gy_ and V_37.5 Gy_) for the rectum plotted against the COPP. These results suggest that the rectum doses in large PTV cases were higher than those in the small PTV cases when the COPP value was the same. Figure 3b,d show the dose‐volume metrics (V_70Gy_ and V_37.5 Gy_) for the rectum plotted against the POPP. These results demonstrate that the POPP appears to correlate with V_70Gy_ and V_37.5 Gy_ more linearly than the COPP does. In terms of the correlation coefficients, the POPP correlated with V_37.5 Gy_ better than the COPP did (*R* = 0.897 and 0.860, respectively) for IMRT, and (*R* = 0.973 and 0.932, respectively) for VMAT. The POPP correlated with V_70Gy_ as well as the COPP (*R* = 0.965 and 0.967, respectively) for IMRT, and (*R* = 0.988 and 0.990, respectively) for VMAT. This result suggests that the predictivity for V_70Gy_ is not improved by considering the PTV volume, and the OAR volume receiving the prescription dose is mainly dependent on the overlap volume. The results of this simulation indicate that the POPP can be effective for predicting rectum dose, especially intermediate dose.

### 3.2 Plan quality and linear regression analysis in simulation 2

The RV and BV were 50.4 ± 6.6 cc and 162.1 ± 29.8 cc, respectively. The overlap ratios (i.e., COPP) for the rectum and bladder were 0.11 ± 0.04 and 0.08 ± 0.02, respectively. The calculated CN in the present study were 0.91 for IMRT, and 0.92 for VMAT. The HI for IMRT and VMAT were 0.065 and 0.088, respectively. These outcomes were better than those reported in other prostate cancer studies.^23^, ^27^ Among the 81 treatment plans generated by Auto‐Planning for IMRT and VMAT, 58 IMRT plans and 63 VMAT plans met the TG119 dose goals.

**FIGURE 4:**
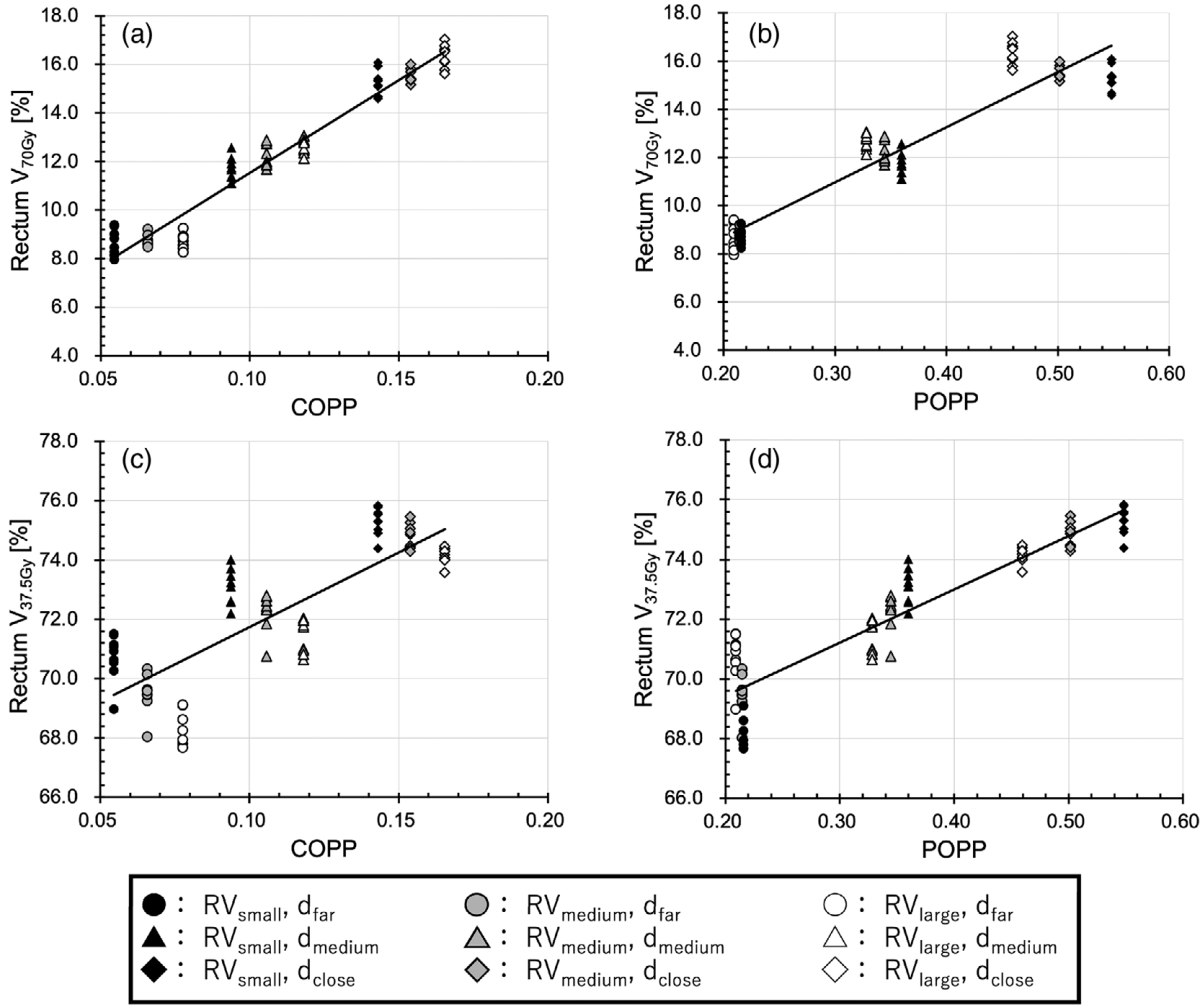
Linear regression analysis of the overlap‐based predictive parameter for rectum V_70Gy_ and rectum V_37.5 Gy_ with various patient cases for IMRT: (a) rectum V_70Gy_ against COPP, (b) rectum V_70Gy_ against POPP, (c) rectum V_37.5 Gy_ against COPP, and (d) rectum V_37.5 Gy_ against POPP. Data are plotted for three types of distances between PTV and rectum: far (circle), medium (triangle), and close (diamond), and for three types of volume: small (black), medium (gray), and large (white). The plot variations in markers of the same shape and same color indicate the result of varying bladder geometry. COPP, conventional overlap‐based predictive parameter; d, distance between rectum and PTV; IMRT, intensity modulated radiation therapy; POPP, proposed overlap‐based predictive parameter; PTV, planning target volume; RV, rectum volume.

**FIGURE 5:**
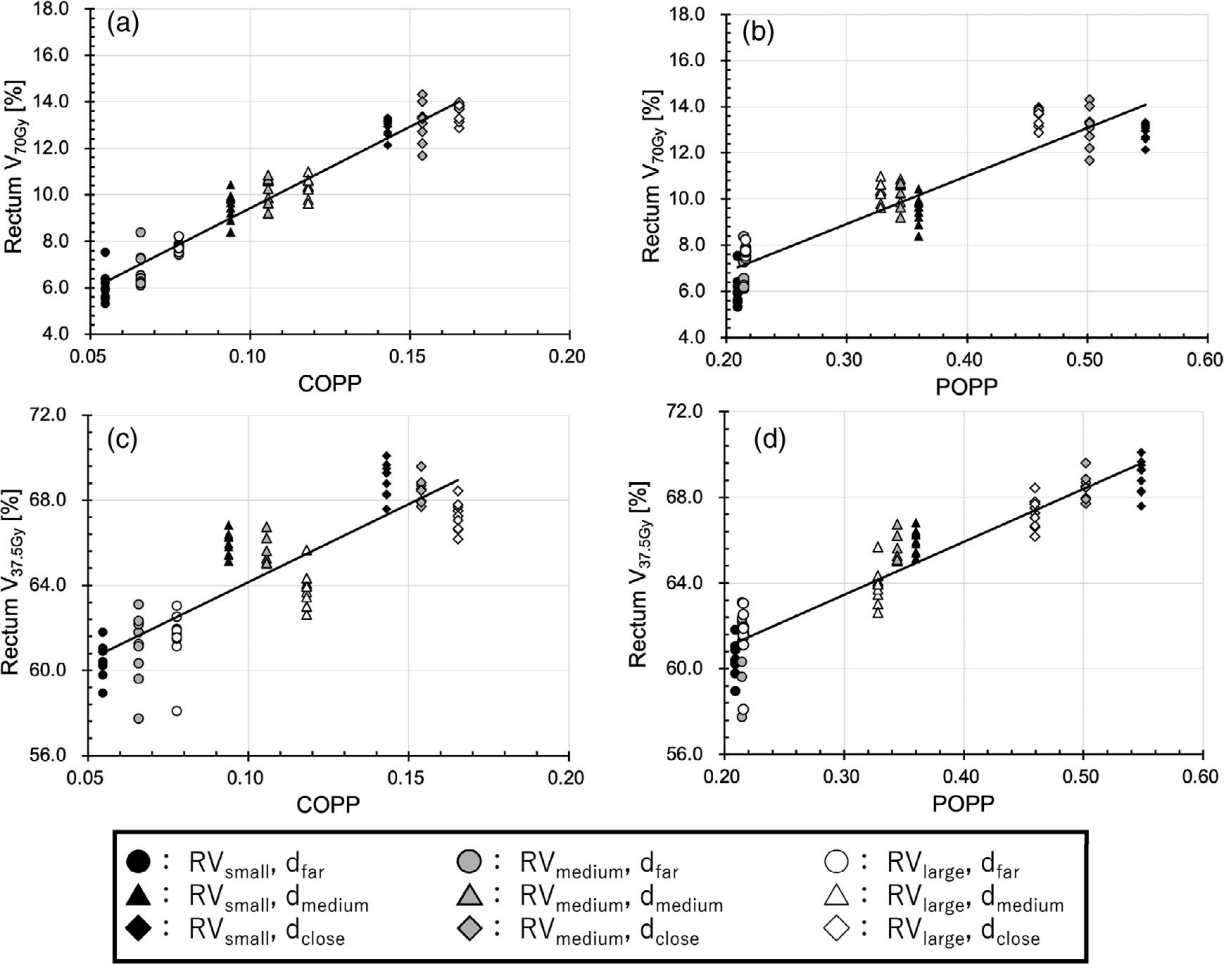
Linear regression analysis of the overlap‐based predictive parameter for rectum V_70Gy_ and rectum V_37.5 Gy_ with various patient cases for VMAT: (a) rectum V_70Gy_ against COPP, (b) rectum V_70Gy_ against POPP, (c) rectum V_37.5 Gy_ against COPP, and (d) rectum V_37.5 Gy_ against POPP. Data are plotted for three types of distances between PTV and rectum: far (circle), medium (triangle), and close (diamond), and for three types of volume: small (black), medium (gray), and large (white). The plot variations in markers of the same shape and same color indicate the result of varying bladder geometry. COPP, conventional overlap‐based predictive parameter; d, distance between rectum and PTV; POPP, proposed overlap‐based predictive parameter; PTV, planning target volume; RV, rectum volume; VMAT, volumetric modulated arc therapy.

Figures 4 and 5 show the dose‐volume metrics (V_70Gy_ and V_37.5 Gy_) plotted against the predictive parameters (COPP and POPP) for IMRT and VMAT, respectively, in simulation 2. The results of dose‐volume metrics and the correlation coefficients between dose‐volume metrics and the predictive parameters are presented in Table 2 for IMRT and in Table 3 for VMAT. In terms of the correlation coefficients, the COPP correlated with high‐dose metrics better than the POPP did, whereas the results also show that the POPP correlated well with intermediate‐dose metrics. These trends were similar to those in simulation 1.

**TABLE 2:** Dose‐volume metrics and correlation coefficients for COPP and POPP in IMRT.

			Correlation coefficient	
OAR	Dose volume metrics		COPP	POPP
Rectum	D_mean_ (Gy)	44.7 ± 1.6	0.865	0.955
	V_70Gy_ (%)	12.2 ± 2.9	0.975	0.945
	V_60Gy_ (%)	24.6 ± 3.6	0.921	0.962
	V_37.5 Gy_ (%)	72.2 ± 2.3	0.806	0.927
Bladder	D_mean_ (Gy)	30.7 ± 1.7	0.870	0.953
	V_70Gy_ (%)	11.1 ± 2.3	0.990	0.802
	V_60Gy_ (%)	15.6 ± 2.7	0.977	0.862
	V_37.5 Gy_ (%)	36.2 ± 3.3	0.881	0.951

Abbreviations: COPP, conventional overlap predictive parameter; IMRT, intensity modulated radiation therapy; POPP, proposed overlap predictive parameter.

**TABLE 3:** Dose‐volume metrics and correlation coefficients for COPP and POPP in VMAT.

			Correlation coefficient	
OAR	Dose volume metrics		COPP	POPP
Rectum	D_mean_ (Gy)	41.4 ± 1.8	0.933	0.964
	V_70Gy_ (%)	10.0 ± 2.7	0.971	0.933
	V_60Gy_ (%)	19.4 ± 3.8	0.946	0.958
	V_37.5 Gy_ (%)	64.8 ± 3.2	0.861	0.940
Bladder	D_mean_ (Gy)	28.5 ± 1.7	0.934	0.924
	V_70Gy_ (%)	9.9 ± 2.3	0.997	0.774
	V_60Gy_ (%)	13.5 ± 2.6	0.993	0.824
	V_37.5 Gy_ (%)	31.2 ± 3.4	0.929	0.934

Abbreviations: COPP, conventional overlap predictive parameter; POPP, proposed overlap predictive parameter; VMAT, volumetric modulated arc therapy.

Simulation 2 indicates that the COPP is effective for predicting high‐dose metrics and that the POPP is effective for predicting intermediate‐dose metrics. We confirmed the dose region, where the COPP or POPP is effective, by comparing the correlation coefficients of the COPP and that of the POPP for dose‐volume metrics in each 1‐Gy increment ranging from V_20Gy_ to V_75Gy_. Our analysis showed a statistically significant superiority of POPP over COPP in predicting rectum doses within the range of V_20Gy_–V_62Gy_ for IMRT and V_20Gy_–V_55Gy_ for VMAT, and bladder doses within the range of V_20Gy_–V_41Gy_ for IMRT and V_20Gy_–V_23Gy_ for VMAT (*p* < 0.05). On the other hand, the COPP had a statistically better correlation than the POPP in rectum doses within the range of V_70Gy_–V_75Gy_ for IMRT and V_68Gy_–V_75Gy_ for VMAT, and bladder doses within the range of V_51Gy_–V_75Gy_ for IMRT and V_42Gy_–V_75Gy_ for VMAT (*p* < 0.05). In this simulation, the threshold dose *Dth*, at which correlation coefficients of the COPP and the POPP for V*_Dth_* were equal, was shown to be 67 Gy for IMRT and 63 Gy for VMAT in the rectum, and 47 Gy for IMRT and 38 Gy for VMAT in the bladder.

## 4 DISCUSSION

In this study, we proposed a POPP and investigated the predictivity of OAR dose using the POPP by comparing the correlation coefficients of POPP and COPP with respect to OAR dose‐volume metrics. The POPP is defined as the COPP multiplied by *V_PTV_/V_OAR_* based on the assumption that the predicted OAR dose should become higher as the PTV volume becomes larger or the OAR volume becomes smaller. The results of the linear regression analysis showed an improvement in the correlation with intermediate‐dose metrics when considering the volume relationship between the PTV and OAR.

As shown in Figures 4a and 5a, the V_70Gy_ for the rectum was found to be about 10% when the COPP was about 0.1. This indicates that the RV receiving more than 70 Gy was nearly equal to the RV overlapping the PTV. In well‐conformed plans, the high‐dose region of the rectum roughly matches the overlap volume. Therefore, the COPP correlated well with the V_70Gy_ for the rectum.

Figures 4b and 5b shows the relationship between the POPP and V_70Gy_ for the rectum. As mentioned above, because V_70Gy_ strongly correlates with the overlap volume, the benefit of modifying the COPP is likely to be limited. The *V_PTV_/V_OAR_* term in Equation (2) makes the POPP smaller as the RV becomes larger. This led to a lower correlation between the POPP and V_70Gy_ for the rectum.

In the low‐dose index case, V_37.5 Gy_ was about 68%, as shown in Figures 4c and 5c, which means that large regions of RV received more than 37.5 Gy. This region largely differed from the overlap volume, as the overlap volume was about 10% in this simulation. Thus, the overlap volume may be insufficient for predicting V_37.5 Gy_. Our simulations confirmed that the correlation between the COPP with V_37.5 Gy_ was lower than that with V_70 Gy_. In terms of the relative volume relationship between PTV and the rectum, large rectum cases can be regarded as relatively small PTV cases. Thus, V_37.5 Gy_ is smaller as the RV becomes larger. This led to a low correlation between COPP and V_37.5 Gy_. On the other hand, as shown in Figures 4d and 5d, POPP correlated well with V_37.5 Gy_, because the *V_PTV_/V_OAR_* term in POPP works to correct the inverted relationship.

It is reported that the high and intermediate doses to rectum and bladder associate with their toxicities.^1^, ^2^, ^3^, ^4^, ^5^, ^6^, ^7^, ^8^, ^9^, ^10^, ^11^, ^12^, ^13^, ^14^ Thus, it is important to control both high and intermediate doses of rectum and bladder. Using COPP and POPP, it is possible to provide the treatment planners with the prediction of high and intermediate dose to OAR before starting dose optimization, which (1) improves treatment efficiency by reducing time to find achievable OAR doses, (2) helps reduce the difference of treatment planning quality among planners, and (3) allows early discussion about planning strategies with physicians in challenging cases.

As shown in Figures 4c and 5c, our results show that the COPP increases but that the V_37.5 Gy_ decreases when the OAR volume becomes larger, resulting in a poor correlation between the COPP and V_37.5 Gy_. With regard to the lower dose‐volume metrics, prior studies reported that the overlap ratio (i.e., COPP) had poor correlation, but intermediate‐dose results were not mentioned in those articles.^21^, ^22^, ^28^ From the clinical data, it is difficult to detect the cause of the poor predictivity for low dose, because OAR sparing is related to various factors such as OAR shapes, the planner's skill, and planning time. In this study, we excluded the dependence on these factors by using Auto‐Planning and systematically varying the phantom volume and position. Our study clarified that the volume relationship between PTV and OAR should be considered for predicting intermediate dose‐volume metrics.

In the recent studies, it is reported that the predictive models for OAR dose have been created using machine learning and the model performance is superior.^29^, ^30^, ^31^ In practical implementation, the utilization of machine learning to predict OAR doses may not be feasible for every institution due to requiring a large number of training data, a development environment for machine learning, and an expertise of machine learning. On the other hand, the process of constructing predictive models for OAR doses using COPP/POPP is straightforward, because this approach solely requires geometric data; the PTV volume, OAR volume, and the overlap volume. Consequently, our proposed methodology has the potential for implementation across a wide array of institutions.

We note the following two limitations of this study:

1. The scope of present study was to propose a new predictive parameter and demonstrate the effectiveness of this parameter. For this purpose, we used a simple phantom in order to simulate systematic anatomical changes including the volume and position between PTV and OAR. However, the structure of human patients are complex than TG119 prostate phantom used in this study. The effectiveness of the POPP for the clinical data will be evaluated using clinical data in future studies.
2. All plans in this study were created using Auto‐Planning. Had the planner manually created the plans, a different result might have been obtained, because the plan includes the planner's variability and intention.

## 5 CONCLUSION

We proposed a POPP that considers the volume relationship between PTV and OAR. Our study showed that the POPP strongly correlated with dose‐volume metrics for low‐intermediate doses, and the COPP had a strong correlation for high doses. Because it has been reported that rectal bleeding and bladder toxicity are associated with low‐intermediate doses as well as high doses, it is important to estimate the dose‐volume metrics across a broad dose range (low‐high doses). The POPP and COPP are suitable for this purpose. This work will be beneficial to help planners to efficiently create the high‐quality treatment plans.

## AUTHOR CONTRIBUTIONS

Yuki Saito, Ryusuke Suzuki, Naoki Miyamoto, and Masaya Tamura collected the data and prepared the materials. Yuki Saito, Ryusuke Suzuki, Naoki Miyamoto, Takahiro Kanehira, Masaya Tamura, Tadashi Mori, Kentaro Nishioka, Takayuki Hashimoto, and Hidefumi Aoyama contributed to the study conception and design. Yuki Saito and Ryusuke Suzuki participated in data analysis. Yuki Saito, Ryusuke Suzuki, Naoki Miyamoto, Kenneth Lee Sutherland, Takahiro Kanehira, and Masaya Tamura wrote the first draft of the manuscript. All authors commented on the manuscript and approved the final manuscript.

## CONFLICT OF INTEREST STATEMENT

The authors declare no conflicts of interest.
